# Exploring the relationship between dysfunctional metacognitive processes and orthorexia nervosa: the moderating role of emotion regulation strategies

**DOI:** 10.1186/s12888-023-05183-z

**Published:** 2023-09-15

**Authors:** Sarah Gerges, Vanessa Azzi, Dora Bianchi, Fiorenzo Laghi, Sara Pompili, Diana Malaeb, Sahar Obeid, Michel Soufia, Souheil Hallit

**Affiliations:** 1 School of Medicine and Medical Sciences, Holy Spirit University of Kaslik, P.O. Box 446, Jounieh, Lebanon; 2 Department of Developmental and Social Psychology, Sapienza University of Rome, Rome, Italy; 3 College of Pharmacy, Gulf Medical University, Ajman, United Arab Emirates; 4 School of Arts and Sciences, Social and Education Sciences Department, Lebanese American University, Jbeil, Lebanon; 5 Applied Science Research Center, Applied Science Private University, Amman, Jordan; 6 Research Department, Psychiatric Hospital of the Cross, Jal Eddib, Lebanon

**Keywords:** Eating disorder, Orthorexia Nervosa, Emotion regulation, Dysfunctional thoughts, Metacognition, Lebanon

## Abstract

**Background:**

Dysfunctional metacognitive processes and emotional dysregulation have been widely documented in the eating disorder literature. Despite numerous research and recent consensus suggesting the categorization of orthorexia nervosa as a form of eating disorder, no previous study has examined whether aberrant metacognitive processes also correlate with orthorexia nervosa tendencies. This paper investigates potential associations between symptoms of orthorexia nervosa and dysfunctional metacognitive processes while also exploring whether such relationships may be influenced by adaptive/maladaptive emotion regulation strategies.

**Methods:**

We conducted a cross-sectional study in all Lebanese governorates. In total, 423 Lebanese adults completed an online questionnaire including the Teruel Orthorexia Scale, the Emotion Regulation Questionnaire, the Difficulties in Emotion Regulation Scale – 16 Item Version, and the Metacognitions Questionnaire – Short Form.

**Results:**

Higher physical activity, expressive suppression, emotion regulation difficulties, positive metacognitive beliefs about worry, and need to control thoughts were significantly associated with higher orthorexia nervosa. Additionally, the emotion regulation strategies moderated the relationships between two dysfunctional metacognitive processes and orthorexia nervosa. Specifically, cognitive self-consciousness was negatively related to orthorexia nervosa only in individuals with low (versus higher) emotion suppression (maladaptive emotion regulation strategy). In contrast, negative beliefs about worry uncontrollability and danger positively predicted orthorexia nervosa only in individuals with lower (versus high) cognitive reappraisal (adaptive emotion regulation strategy). Lower socio-economic status and having a university level of education compared to secondary level were significantly associated with lower orthorexia nervosa.

**Conclusion:**

Our study provides the first empirical evidence for the existence of significant associations between dysfunctional metacognitive processes and orthorexia nervosa. It also highlights that these pathways are considerably modulated by individuals’ ability to regulate their emotions adaptively versus maladaptively. Our findings thus suggest that therapies aimed at improving thought monitoring and emotional regulation may be beneficial for individuals with symptoms of orthorexia nervosa.

## Introduction

Over recent decades, societies have been placing a growing emphasis on the importance of healthy eating, to the point that discussions regarding the ideal type, timing, and quantity of food consumption have increasingly become a significant part of social discourse and judgment [1]. As a result, researchers have witnessed the emergence of a novel entity labelled “Orthorexia Nervosa”, which was first introduced by Bratman in 1997 [2]. Orthorexia nervosa derived from the Greek terms “Ortho” (i.e., righteous) and “Orexia” (i.e., appetite), thus alluding to an unhealthy obsession with clean and healthy eating [3, 4] whose ultimate goal is to achieve purity and health [5]. Individuals with orthorexia nervosa tend to exponentially devote their time to activities related to meal planning and consumption, which interferes with various life aspects; to exemplify, they may avoid social situations involving eating out with others and even experience physical complications such as extreme weight loss and malnutrition [6, 7]. Despite an emerging body of research attempting to determine its classification and diagnostic criteria, this entity has neither been assigned a standardized definition nor included in the Diagnostic and Statistical Manual of Mental Disorders (DSM-5-TR) [8] or any other international disease classification [9, 10].

While orthorexia nervosa’s overlap with Obsessive-Compulsive Disorder (OCD) in terms of obsessive characteristics, intrusive thoughts, and repetitive behaviors is still controversial [11–14], orthorexia nervosa is specifically centered around an obsession with food choices, healthy eating, and strict dietary habits [15, 16]. In contrast, OCD encompasses a broader spectrum of obsessions and compulsions unrelated to food, such as cleanliness, symmetry, or fears of harm [8]. Furthermore, the underlying motivations and beliefs differ, as orthorexia nervosa is driven by a pursuit of health and wellness, while OCD is characterized by irrational fears and the need to alleviate anxiety through repetitive actions [17, 18]. Numerous papers have however indicated that orthorexia nervosa also shares similarities with some eating disorders, such as anorexia nervosa and bulimia nervosa [19], and may even arise during the post-recovery phase of an eating disorder [20]. Symptoms of orthorexia may also frequently mask symptoms of other eating disorders, such as anorexia nervosa; in fact, in clinical settings, the simultaneous presence of orthorexia nervosa alongside other well-established eating disorders is commonly observed [21, 22]. A recent consensus document on the definition and diagnostic criteria for orthorexia nervosa has also suggested its categorization as a mental health disorder falling within the DSM-5’s “Feeding and Eating Disorders” section (with 93.3% of experts agreement), indicating that it could be identified as a distinct form of eating disorder [9]. Nonetheless, further research remains vital to determine whether orthorexia nervosa is similar to or distinct from other types of eating disorders, as well as to deeply scrutinize its features and predictors, in order to gain a better understanding of this condition’s psychopathology and hence develop effective treatments.

In this context, a potentially crucial aspect that has been severely overlooked in research is whether dysfunctional metacognitive processes may contribute to the induction and/or perpetuation of orthorexia nervosa symptoms. Metacognition refers to the ability to reflect on and monitor one’s own thinking processes; it is described as a resolutive cognitive strategy involving multiple psychological aspects, such as perception, control, examination, and adjustment of an individual’s thinking processes [23]. Dysfunctional metacognitive processes, on the other hand, are maladaptive patterns of thinking often leading to negative psychological and behavioral outcomes [23, 24]. They are mainly classified into positive and negative metacognitive beliefs. As such, positive beliefs are postulated to serve as evaluative mechanisms that assess the efficacy of certain cognitive processes (e.g., beliefs in the effectiveness of anxiety and worrying thoughts, excessive cognitive self-consciousness, etc.), with the goal of guiding and adjusting one’s thoughts and emotions; whereas negative beliefs involve a pervasive conviction of insufficient control over one’s cognitive and affective states (e.g., worry uncontrollability and danger, low cognitive confidence, need to control thoughts, etc.); both potentially leading to maladaptive patterns of coping, thinking, and behaving [23–25]. As for disordered eating behaviors, these dysfunctional metacognitive processes, often involving ruminative ideation and excessive worry, may give rise to heightened preoccupations with food and eating behaviors [26, 27]. Indeed, dysfunctional metacognitive processes have been extensively documented in the eating disorder literature [26, 28–31]. Nevertheless, no previous research has examined whether maladaptive metacognitive thoughts also correlate with orthorexia nervosa tendencies.

Moreover, emotion regulation difficulties and deficient emotion regulation strategies are important constructs that have been consistently demonstrated to be intricately related to eating disorders [32–36]. Emotion regulation refers to the use of cognitive and behavioral strategies to influence the emotional expression or response, with the aim of achieving a desired emotional state or outcome [37]. Such proficiency is essential for effectively managing aversive emotional experiences and requires several key cognitive-behavioral assets, namely a high level of emotional comprehension, awareness, and acceptance without judgment or avoidance; as well as the aptitude to engage in goal-directed behaviors, exercise impulse control, and use proficient emotion regulation strategies [38]. Alterations of any of these cognitive dimensions (i.e., emotion regulation difficulties) may contribute to the development and/or perpetuation of eating disorders as a maladaptive means to cope with negative emotions, and have been frequently observed in adults with such disorders compared to controls [33, 39, 40], as well as recently in orthorexia nervosa [41, 42].

On another hand, since emotion regulation implies the use of various approaches contingent on the stage and timing within the emotion production process [43], two emotion regulation strategies may be potentially enacted in response to adverse emotional experiences: expressive suppression and cognitive reappraisal [44, 45]. Expressive suppression is a response-focused strategy often viewed as maladaptive, as it involves suppressing or reducing the behavioral and physiological expressions of ongoing negative emotions without circumventing their inner experience by the individual [37, 43, 46]. In contrast, cognitive reappraisal is an antecedent-focused emotion regulation strategy often seen as adaptive, as it interferes at the initial stages of emotional processing by modifying the perception and interpretation of an emotion-eliciting situation, hence preventing the development of negative emotions as well as their deleterious effects on individuals’ emotional experiences [37, 43, 46, 47]. Empirical investigations on the frequent use of expressive suppression as an emotion regulation strategy have consistently demonstrated its detrimental impact on various aspects of psychological well-being, as individuals experience unresolved aversive emotions and a feeling of discrepancy between their emotional experiences and behavioral expressions, potentially leading them to psychopathological issues including eating disorders [47–52]; whereas the opposite has been remarked for cognitive reappraisal, with no or even inverse relationships with eating disorder symptoms [51–53]. However, to the best of our knowledge, no study has attempted to explore the different relationships between expressive suppression/cognitive reappraisal and orthorexia nervosa to date.

In addition, newly emerging empirical evidence interestingly suggests the existence of a potential interplay between dysfunctional metacognitive beliefs and compromised emotion regulation capacities, which may act in concert to predict the onset and maintenance of various forms of psychopathology, including disordered eating behaviors [54–57]. In fact, since dysfunctional metacognitive appraisals serve as a mechanism for maladaptive ruminative thought processes and worry, it is posited that they also interfere in regulating emotions, thus frequently giving rise to overall deficits in emotion regulation and emotional difficulties, and resulting in heightened psychological issues and distress [24, 58, 59]. Indeed, the Self-Regulatory Executive Function model (S-REF) [59, 60] proposes a bidirectional causal relationship between dysfunctional metacognitive processes and emotional dysregulation, highlighting the influential role of dysfunctional metacognitive processes in the development of emotional disorder symptoms and psychopathological symptoms by fostering prolonged negative thinking in response to stress. Mutually, the model acknowledges the potential impact of emotions on metacognition, as emotion dysregulation may for instance reinforce dysfunctional metacognitive beliefs related to a perceived loss of control [59–61]. In line with this perspective, it is plausible to conjecture that deficient emotion regulation strategies (i.e., expressive suppression) and emotion regulation difficulties may positively moderate/strengthen the relationships between dysfunctional metacognitive processes and eating disorder symptoms like orthorexia nervosa tendencies, by affecting the extent to which people with orthorexia nervosa rely on food and eating behaviors to cope with negative emotions. Since individuals experiencing emotion regulation difficulties are particularly vulnerable to using food and eating behaviors as a means of regulating negative affect [62], ameliorations of negative emotions subsequent to indulging in such behaviors may reinforce these maladaptive strategies and potentially exacerbate the distorted thought patterns associated with dysfunctional metacognitive processes (i.e., excessive rumination, perceived loss of control, and worry towards eating patterns), thereby contributing to the maintenance and exacerbation of the eating disorder symptoms [27, 63]. Alternatively, it is also conceivable that the utilization of effective emotion regulation strategies, such as cognitive reappraisal, may negatively moderate/alleviate the impact of dysfunctional metacognitive beliefs on the development or exacerbation of orthorexia nervosa. By efficiently managing negative emotions linked to maladaptive metacognitive appraisals, individuals may possess higher capability to refrain from partaking in disordered food-related behaviors [51, 52] such as orthorexia nervosa tendencies. Nevertheless, no empirical investigations have been conducted to evaluate these postulations.

To this end, the current study aims to address the aforementioned gaps in the literature by examining the potential associations between symptoms of orthorexia nervosa and dysfunctional metacognitive processes, as well as both emotion regulation difficulties and strategies. Specifically, our study seeks to explore whether adaptive (cognitive reappraisal) and/or maladaptive (expressive suppression) emotion regulation strategies may influence the relationships between dysfunctional metacognitive processes and orthorexia nervosa. We hypothesize that, in addition to dysfunctional metacognitive processes and emotion regulation difficulties, only emotion suppression will be positively associated with orthorexia nervosa and reinforce the relationships between dysfunctional thought processes and orthorexia nervosa. Conversely, cognitive reappraisal is expected to mitigate such associations.

## Methods

### Participants

The sample consisted of 423 participants, with a mean age of 38.13 ± 11.03 years and 61.2% women. Other characteristics and description of the scores can be found in Table 1.

**Table 1:** Sociodemographic characteristics of the participants (N = 423).

Variable	N (%)
**Gender**	
Men	164 (38.8%)
Women	259 (61.2%)
**Marital status**	
Single/divorced/widowed	151 (35.7%)
Married	272 (64.3%)
**Education level**	
Secondary or less	54 (12.8%)
University	369 (87.2%)
**Religion**	
Christian	174 (44.6%)
Muslim	216 (55.4%)
	**Mean ± SD**
Age (in years)	38.13 ± 11.03
Body Mass Index	26.39 ± 5.18
Household crowding index	0.92 ± 0.54
Physical activity index	24.34 ± 19.52
TOS Orthorexia nervosa	14.71 ± 4.98
Low cognitive confidence	13.09 ± 4.57
Positive beliefs about worry	13.57 ± 5.32
Cognitive self-consciousness	17.49 ± 3.91
Negative beliefs about worry uncontrollability and danger	13.13 ± 5.04
Need to control thoughts	13.76 ± 4.26
DERS total score	48.36 ± 14.55
Expressive suppression	32.92 ± 20.84
Cognitive reappraisal	5.89 ± 4.70

TOS = Teruel Orthorexia Scale, DERS = Difficulties in Emotion Regulation Scale

### Study design and procedure

We conducted a cross-sectional study between June and July 2021 in all Lebanese governorates (Beirut, Mount Lebanon, North, South, and Bekaa). Due to the coronavirus pandemic outbreak, the data were gathered through snowball sampling using an online questionnaire. Prior to participation, study objectives and general instructions were delivered online for the individual subjects. We restricted multiple submissions from the same responder by using the “Restrict to 1 response” setting on Google Forms, where all IP addresses were examined. Participation was voluntary and anonymous. No credits were received for participation.

### Minimal sample size calculation

According to the G-power software [64], 371 participants were needed to have enough statistical power, based on a minimal deviation of R^2^ from zero of 5%, a 5% risk of error, a 80% power, and 10 factors to be entered in the multivariate analyses.

### Questionnaire and measures

The questionnaire was divided into three parts. In the first part, a written consent, confirming the approval of the participant to fill in the questionnaire was gathered. In the second part, respondents answered to questions assessing socio-demographic details (age, residency governorate, height, weight, etc.). The Household Crowding Index (HCI), reflecting the socio-economic status of the family, was calculated by dividing the number of people living in the house by the number of rooms in the house; a higher HCI reflects a lower socio-economic status [65]. In the last part of the study, participants completed a set of self-report measures, as follows:

#### The teruel orthorexia scale

The Teruel Orthorexia Scale (TOS), validated in Lebanon [66, 67], is a 17-item instrument that assesses orthorexia nervosa with two separate dimensions [68]: 9 items for Healthy Orthorexia or “HeOr” (e.g., “I mainly eat foods that I consider healthy”) and 8 items for Orthorexia Nervosa or “OrNe” (e.g., “Thoughts about healthy eating do not let me concentrate on other tasks”). Responses are provided on a four-point Likert-type scale ranging from 0 = “strongly disagree” to 3 = “strongly agree”. Scores by dimension were computed as the sum of the item responses. In this study, the internal consistencies were ω = 0.85 for the TOS OrNe and ω = 0.85 for the TOS HeOr. In this paper, the TOS OrNe score were used.

#### The difficulties in emotion regulation scale – 16 Item Version (DERS-16)

It is a 16-item scale that assesses emotion regulation [69]. Items are graded using a 5-point Likert scale. Higher scores reflect increased emotion regulation difficulties. Within the scale are five subscales: emotional clarity (2 items; e.g., “I am confused about how I feel”), goals (3 items; e.g., “When I am upset, I have difficulty thinking about anything else”), impulse (3 items; e.g., “When I am upset, I feel out of control”), non-acceptance (3 items; e.g., “When I am upset, I feel like I am weak”), and strategies (5 items; e.g., “When I am upset, I believe that there is nothing I can do to make myself feel better”). The total score was used in this study (ω = 0.95). We used the validated Arabic version of this scale [70].

#### The emotion regulation questionnaire (ERQ)

Validated in Lebanon [71], it is a ten-item scale, with questions scored on a 7-point Likert scale [72]. Two dimensions derive from this scale: Expressive Suppression (e.g., “I control my emotions by not expressing them”) and Cognitive Reappraisal (e.g., “I control my emotions by changing the way I think about the situation I’m in”). Higher cognitive reappraisal scores are considered a positive/adaptive emotion regulation strategy (ω = 0.81), whereas higher emotional suppression is considered maladaptive within emotion regulation strategies (ω = 0.72).

#### The metacognitions questionnaire short form (MCQ-30)

Validated in Arabic [73], this tool [23] was used to assess dysfunctional metacognition processes. It consists of five dimensions, each composed of 6 items rated on a 4-point Likert scale from 1 (“Disagree”) to 4 (“Agree”): cognitive confidence, which concerns the beliefs a person holds about their cognitive abilities, including memory and attention (e.g., “I do not trust my memory”); positive beliefs about worry, which describes worrying as a problem-solving technique (e.g., “I need to worry in order to work well”); cognitive self-consciousness, which evaluates the tendency to constantly focus attention on one’s own thinking processes (e.g., “I constantly examine my thoughts”); negative beliefs about worry uncontrollability and danger, which describes worry as being uncontrollable and dangerous (e.g., “My worrying thoughts persist, no matter how I try to stop them”); and need to control thoughts, which assesses the belief of the person about thoughts having to be controlled/suppressed (e.g., “If I could not control my thoughts, I would not be able to function”). The McDonald’s ω values for the subscales were as follows: cognitive confidence (= 0.86), positive beliefs about worry (= 0.94), cognitive self-consciousness (= 0.84), negative beliefs about worry uncontrollability and danger (= 0.89) and need to control thoughts (= 0.78).

### Statistical analyses

Since the data were collected using an online questionnaire, there were no missing values because responding to all questions was required. SPSS v.25 was used to compute data analyses. Prior to the analyses, normality of distribution of the TOS score was confirmed via a calculation of the skewness and kurtosis. Values between − 2 and + 2 are considered acceptable to prove normal univariate distribution [74]. These conditions consolidate the assumptions of normality in samples larger than 300 [75]. The scales’ internal consistency was evaluated using McDonald’s ω, and a threshold of 0.70 or higher was considered indicative of acceptable internal consistency [76]. McDonald’s ω was chosen as an alternative measure to Cronbach’s alpha due to concerns raised about the limitations associated with using Cronbach’s alpha [77]. Pearson’s test was used to correlate the TOS score with other continuous variables, whereas the Student’s t-test was used to compare two means. A set of linear regressions taking the TOS OrNe score as the dependent variable, was conducted as follows: in Step 1, socio-demographic variables were entered as covariates; in Step 2, DERS, Cognitive reappraisal, and Expressive suppression scores were entered, whereas in Step 3 the MCQ dimensions were entered. The PROCESS Macro v. 3.4 model 1 was then used to estimate significant interactions between each MCQ subscale score, taken as independent variables, and DERS and ERQ dimensions, taken as moderator variables. All variables were preliminarily standardized before computing interactions. Covariates were only entered in the linear regression if they were to show a *p* < .25 in the bivariate analysis [78]. A *p* < .05 was deemed statistically significant.

## Results

### Bivariate analysis

The bivariate analysis results are summarized in Tables 2 and 3. Higher cognitive reappraisal, expressive suppression, and physical activity index were significantly associated with higher orthorexia nervosa, whereas higher household crowding index was significantly associated with lower orthorexia nervosa. Lower cognitive confidence (i.e., higher scores on the cognitive confidence subscale) and increased positive beliefs about worry, negative beliefs about worry uncontrollability and danger, and need to control thoughts were significantly associated with higher orthorexia nervosa. Lower cognitive confidence and increased need to control thoughts were significantly associated with higher expressive suppression, whereas higher cognitive self-consciousness was significantly associated with higher cognitive reappraisal. On another hand, participants with a secondary level of education or less significantly had a higher mean orthorexia nervosa score compared to those who had a university education level.

**Table 2:** Correlation between orthorexia nervosa and other continuous variables

Variable	1	2	3	4	5	6	7	8	9	10	11	12	13
1. Orthorexia nervosa	1												
2. Cognitive confidence	0.13^**^	1											
3. Positive beliefs about worry	0.29^***^	0.35^***^	1										
4. Cognitive self-consciousness	0.07	0.03	0.31^***^	1									
5. Negative beliefs about worry uncontrollability and danger	0.21^***^	0.54^***^	0.56^***^	0.35^***^	1								
6. Need to control thoughts	0.21^***^	0.40^***^	0.40^***^	0.30^***^	0.68^***^	1							
7. DERS total score	− 0.08	− 0.53^***^	− 0.34^***^	0.04	− 0.60^***^	− 0.60^***^	1						
8. Expressive suppression	0.16^**^	0.17^**^	0.05	0.07	0.16^**^	0.17^**^	0.29^***^	1					
9. Cognitive reappraisal	− 0.11^*^	− 0.03	− 0.05	− 0.18^***^	− 0.03	− 0.15^**^	0.05	− 0.62^***^	1				
10. Age	− 0.03	0.01	− 0.001	0.05	− 0.08	− 0.06	0.07	0.05	0.23^***^	1			
11. Body Mass Index	− 0.07	0.08	− 0.09	− 0.13*	− 0.09	− 0.01	− 0.17^**^	0.18^***^	0.14^**^	0.12^*^	1		
12. Household crowding index	− 0.15^**^	− 0.07	0.03	0.08	0.03	0.02	0.07	− 0.17^***^	− 0.09	0.03	− 0.28^***^	1	
13. Physical activity index	0.20^***^	− 0.17^***^	0.01	− 0.08	− 0.06	− 0.05	0.16^**^	− 0.02	− 0.15^**^	− 0.25^***^	− 0.07	− 0.21^***^	1

Notes: ***p &lt; 0.001; **p &lt; 0.01; *p &lt; 0.05 ; DERS = Difficulties in Emotion Regulation Scale;

**Table 3:** Group differences by categorical variables on orthorexia nervosa mean scores

Variable	Mean ± SD	*T*	*df*	*p*
**Gender**		-1.36	420	0.173
Male	15.96 ± 4.96			
Female	16.68 ± 5.74			
**Marital status**		1.83	420	0.068
Single/divorced/widowed	16.99 ± 6.12			
Married	15.97 ± 4.90			
**Education level**		3.63	420	**< 0.001**
Secondary or less	16.85 ± 5.49			
University	14.41 ± 4.86			
**Religion**		0.70	406	0.484
Christian	16.71 ± 5.83			
Muslim	16.33 ± 5.16			

Numbers in bold indicate significant p-values.

### Multivariate analyses

The results of a linear regression, taking the orthorexia nervosa score as the dependent variable, showed that higher physical activity index (Beta = 0.01), expressive suppression (Beta = 0.14), emotion regulation difficulties (Beta = 0.20), positive metacognitive beliefs about worry (Beta = 0.22), and need to control thoughts (Beta = 0.20) were significantly associated with higher orthorexia nervosa, whereas lower socio-economic status (i.e., higher household crowding index) (Beta=-0.27) and having a university level of education compared to secondary level (Beta=-0.29) were significantly associated with lower orthorexia nervosa (Table 4).

**Table 4:** Linear regression analysis taking the orthorexia nervosa score as the dependent variable

	Unstandardized Beta	Standardized Beta	*p*	95% CI
**Step 1: Covariates as independent variables**				
Body Mass Index	-0.02	-0.12	**0.016**	-0.03; -0.004
Household crowding index	-0.34	-0.17	**0.001**	-0.53; -0.14
Physical activity index	0.01	0.16	**0.002**	0.003; 0.01
Marital status (married vs. single*)	-0.16	-0.08	0.088	-0.35; 0.03
Education (university vs. secondary*)	-0.51	-0.20	**< 0.001**	-0.75; -0.27
Gender (females vs. males*)	0.09	0.04	0.365	-0.11; 0.29
**Step 2: Covariates, DERS, cognitive reappraisal, and expressive suppression as independent variables**				
Body Mass Index	-0.02	-0.14	**0.006**	-0.04; -0.01
Household crowding index	-0.28	-0.15	**0.006**	-0.48; -0.08
Physical activity index	0.01	0.19	**< 0.001**	0.004; 0.01
Marital status (married vs. single*)	-0.13	-0.07	0.171	-0.32; 0.06
Education (university vs. secondary*)	-0.43	-0.17	**0.001**	-0.68; -0.19
Gender (females vs. males*)	0.11	0.05	0.303	-0.10; 0.31
Emotion regulation difficulties (DERS)	0.06	0.06	0.270	-0.04; 0.16
Cognitive reappraisal	-0.09	-0.09	0.152	-0.21; 0.03
Expressive suppression	0.06	0.06	0.328	-0.07; 0.19
**Step 3: Covariates, DERS, cognitive reappraisal, expressive suppression, and MCQ subscales as independent variables**				
Body Mass Index	-0.01	-0.08	0.128	-0.03; 0.003
Household crowding index	-0.27	-0.14	**0.007**	-0.46; -0.07
Physical activity index	0.01	0.16	**0.001**	0.003; 0.01
Marital status (married vs. single*)	-0.06	-0.03	0.535	-0.24; 0.13
Education (university vs. secondary*)	-0.29	-0.11	**0.022**	-0.54; -0.04
Gender (females vs. males*)	0.15	0.08	0.130	-0.05; 0.35
Emotion regulation difficulties (DERS)	0.20	0.20	**0.005**	0.06; 0.34
Cognitive reappraisal	-0.02	-0.02	0.768	-0.14; 0.11
Expressive suppression	0.14	0.14	**0.035**	0.01; 0.27
Cognitive confidence	0.04	0.04	0.512	-0.07; 0.15
Positive beliefs about worry	0.22	0.22	**< 0.001**	0.11; 0.33
Cognitive self-consciousness	-0.09	-0.09	0.122	-0.20; 0.02
Negative beliefs about worry uncontrollability and danger	0.05	0.05	0.529	-0.10; 0.20
Need to control thoughts	0.20	0.20	**0.004**	0.06; 0.33

*Reference group; numbers in bold indicate significant p-values. Nagelkerke R^2^ values: model 1: 11.1%; model 2: 13.3%; model 3: 20.9%

### Interaction analyses

**Fig. 1:**
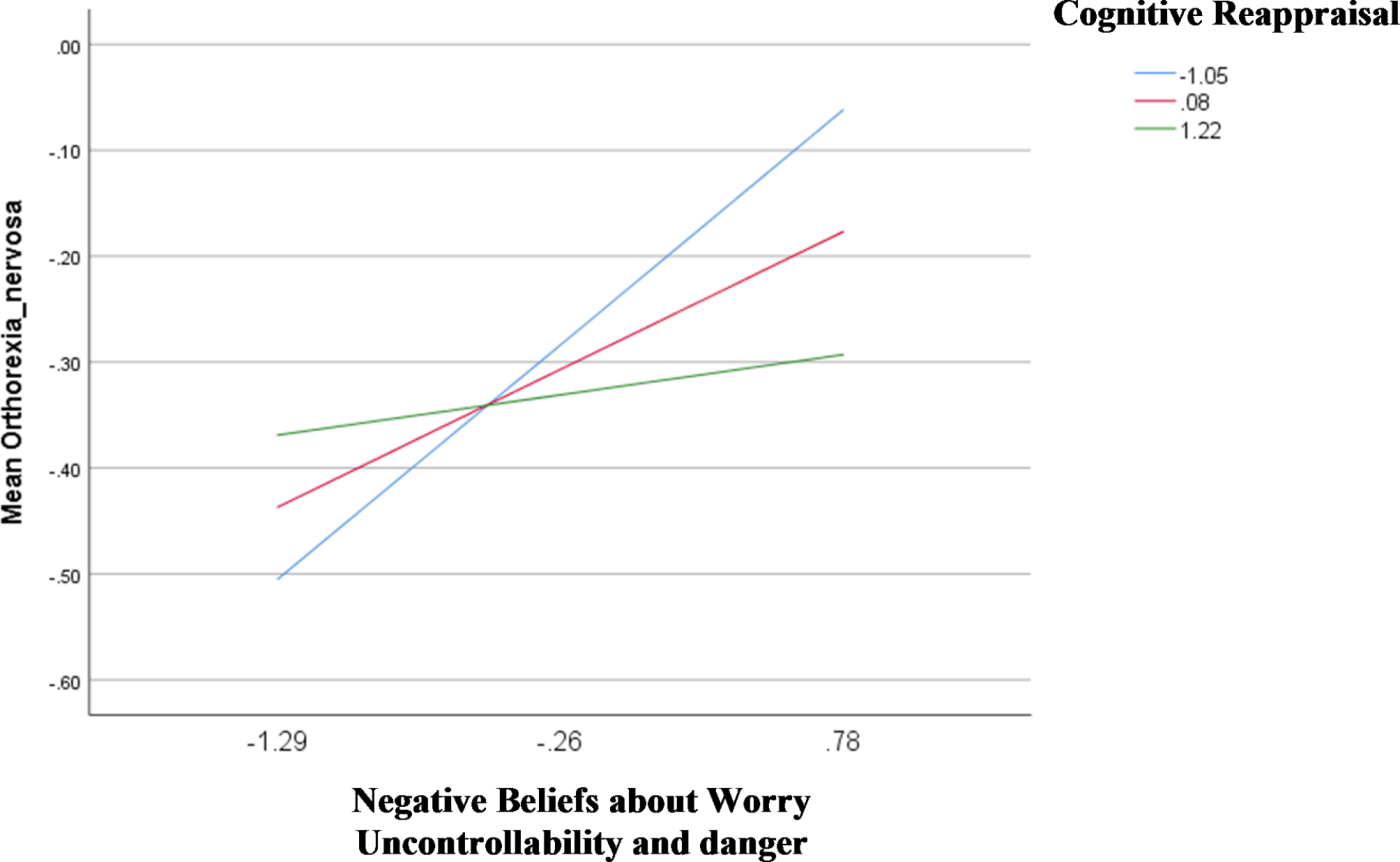
Interaction negative beliefs about worry uncontrollability and danger by cognitive reappraisal on orthorexia nervosa

**Fig. 2:**
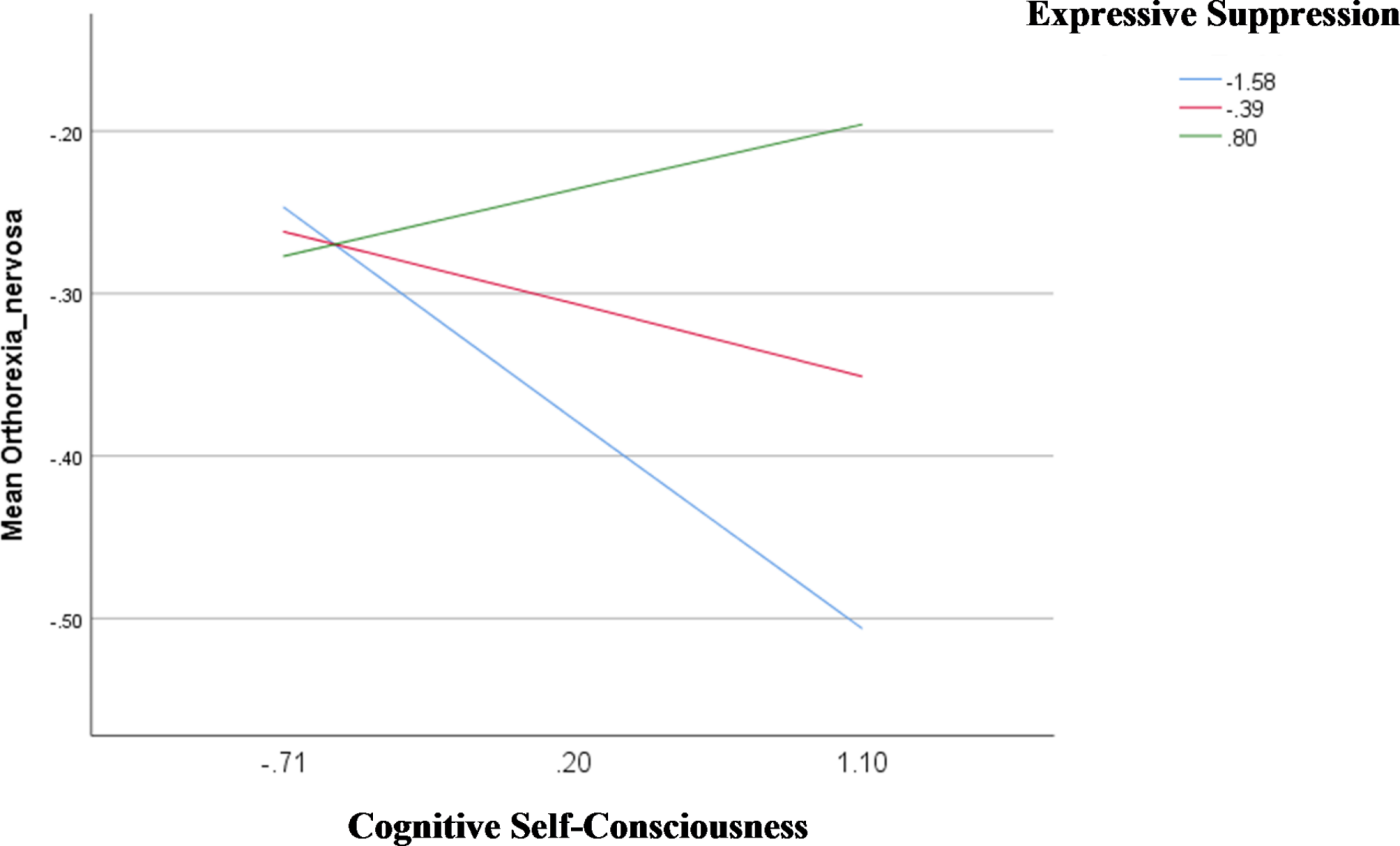
Interaction cognitive sef-consciousness by expressive suppression on orthorexia nervosa

The results of the interaction analyses are summarized in Table 5 (Table 5). The interactions between (1) negative beliefs about worry uncontrollability and danger by cognitive reappraisal (Fig. 1), and (2) cognitive self-consciousness by expressive suppression (Fig. 2) were significantly associated with orthorexia nervosa; at low (Beta = 0.21; *p* = .001) and moderate (Beta = 0.13; *p* = .029) levels of cognitive reappraisal, having higher negative beliefs about worry uncontrollability and danger was significantly associated with higher orthorexia nervosa (Table 6). Furthermore, at low levels of expressive suppression, higher levels of cognitive self-consciousness (Beta = − 0.14; *p* = .039) were significantly associated with lower orthorexia nervosa (Table 7).

**Table 5:** Moderation analyses: MCQ subscales as independent variables, ERQ and DERS as moderators, orthorexia as dependent variable

	Beta	*t*	*p*	95% CI
**Model 1: Cognitive reappraisal as the moderator**				
Cognitive confidence	-0.02	-0.58	0.562	-0.09; 0.05
Positive beliefs about worry	0.002	0.06	0.952	-0.07; 0.07
Cognitive self-consciousness	-0.05	-1.35	0.179	-0.12; 0.02
Negative beliefs about worry uncontrollability and danger	-0.08	-2.27	**0.024**	-0.15; -0.01
Need to control thoughts	-0.06	-1.72	0.085	-0.12; 0.01
**Model 2: Expressive suppression as the moderator**				
Cognitive confidence	-0.01	-0.20	0.840	-0.07; 0.06
Positive beliefs about worry	0.02	0.49	0.623	-0.05; 0.09
Cognitive self-consciousness	0.08	2.06	**0.040**	0.004; 0.15
Negative beliefs about worry uncontrollability and danger	0.04	1.31	0.191	-0.02; 0.11
Need to control thoughts	0.01	0.14	0.889	-0.06; 0.07
**Model 3: Difficulties in emotion regulation as the moderator**				
Cognitive confidence	-0.03	-0.67	0.503	-0.11; 0.05
Positive beliefs about worry	-0.02	-0.39	0.695	-0.10; 0.07
Cognitive self-consciousness	0.08	1.77	0.078	-0.01; 0.17
Negative beliefs about worry uncontrollability and danger	-0.02	-0.62	0.538	-0.10; 0.05
Need to control thoughts	-0.05	-1.36	0.173	-0.13; 0.02

Standardized scores were used in this analysis; numbers in bold indicate significant *p* values

**Table 6:** Conditional effects of the focal predictor (negative beliefs about worry) at values of the moderator (cognitive reappraisal)

	Beta	*T*	*p*	95% CI
Low (= -1.05)	0.21	3.20	**0.001**	0.08; 0.35
Moderate (= 0.08)	0.13	2.20	**0.029**	0.01; 0.24
High (= 1.22)	0.04	0.52	0.603	− 0.10; 0.18

Numbers in bold indicate significant *p* values.

**Table 7:** Conditional effects of the focal predictor (cognitive self-consciousness) at values of the moderator (expressive suppression)

	Beta	*T*	*p*	95% CI
Low (= -1.58)	− 0.14	-2.07	**0.039**	− 0.28; − 0.01
Moderate ( = − 0.39)	− 0.05	-1.01	0.314	− 0.15; 0.05
High (= 0.80)	0.04	0.70	0.487	− 0.08; 0.17

Numbers in bold indicate significant *p* values.

## Discussion

In this paper, our main objectives were to explore the relationships between dysfunctional metacognitive processes and orthorexia nervosa, as well as to investigate the potential role of adaptive (cognitive reappraisal) and/or maladaptive (expressive suppression) emotion regulation strategies within these associations. Our results provided novel insights into the metacognitive correlates of orthorexia nervosa. The analysis has indeed indicated that the need to control thoughts and positive metacognitive beliefs about worry were significantly related to increased orthorexia nervosa tendencies. These findings are in line with previous literature describing dysfunctional metacognitive processes in eating disorders [30]. For instance, individuals with eating disorders commonly display elevated levels of positive metacognitions regarding the efficacy of anxious and worrying thoughts [30]. Specifically, these metacognitions pertain to the anguish of gaining weight and the desire to maintain a specific body shape, which are also generally observed in people with eating disorders [79–81]. In the case of orthorexia nervosa, individuals may instead exhibit preoccupations with the fear of consuming an unhealthy diet and causing harm to their health, which can lead to an irrational and debilitating fixation on consuming only healthy foods.

Furthermore, the need to control thoughts has also been consistently established as a susceptibility factor for dysfunctional eating behaviors [26, 30, 82, 83]. Namely, individuals are inclined to engage in detrimental eating patterns in furtherance of regulating their thoughts, compensating for their low perception of control over internal emotions and external circumstances (a key cognitive factor in eating disorders) [84], and hence, evading unfavorable consequences. Resorting to disordered eating behaviors thus reflects their desire of perceiving some degree of self-control [84]. The current study thus speculates that this phenomenon may also be observed within the context of orthorexia nervosa, whereby individuals exhibit a pattern of restrictive eating behaviors that is characterized by the consumption of only foods deemed “healthy”, mainly as a means of sensing a subjective control over their health. Moreover, since people engaging in maladaptive eating behaviors commonly display a strong preoccupation with regulating and suppressing thoughts pertaining to their body weight and shape, this persistent focus leads to an even more inflated attentiveness to these body concerns, which may in turn contribute to maintaining the eating disorder [56]. In line with the previous statement, it has also been demonstrated that orthorexic behaviors can progressively intensify over time [85], contributing to the maintenance and chronicity of the disorder.

Additionally, our study demonstrated that emotion regulation difficulties and maladaptive emotion regulation strategies (i.e., expressive suppression) were significantly associated with orthorexia nervosa among Lebanese adults, consistent with previous findings [41, 42]. Precisely, people with orthorexia nervosa showed a tendency to reject emotional responses, employ limited and unsuitable strategies for regulating their emotions, and experience difficulties in adopting goal-directed behaviors and controlling impulsive actions when experiencing distress [41]. Researchers suggested that when individuals are persuaded of the lack of alternative strategies that can effectively improve their emotional well-being [86, 87] (observed with deficient emotion regulation strategies), extreme and obsessive orthorexic eating behaviors may offer a sense of control over their emotions, thereby serving as a coping strategy to manage difficult emotional experiences [41]. Inappropriate self-imposed restrictive eating may then provide a feeling of security and reassurance over threatening situations and adverse emotional states, such as self-criticism related to body shape [88, 89] or unhealthy eating in the case of orthorexia nervosa. In this context, emotion regulation difficulties and expressive suppression have been documented across the broad spectrum of eating disorders as well [39, 40, 50, 51].

Remarkably, and as hypothesized, our analyses revealed that adaptive and maladaptive emotion regulation strategies exert key moderating effects in the relationships between dysfunctional metacognitive processes and orthorexia nervosa. Prior research has observed that in response to negative emotional expressions, people endeavor to regulate their thoughts [82], particularly the cognitive manifestations of underlying struggles with coping with distress [26]. This suggests that when emotion regulation strategies are deficient in coping with internal distress, dysfunctional metacognitive beliefs arise and fail to be counteracted, hence resulting in compensatory psychopathological disorders (e.g., eating disorders). Additional research has also emphasized the complex interplay between metacognitive and emotional processes in contributing to disordered eating behaviors [55–57].

In particular, among those with higher negative beliefs about worry uncontrollability and danger, participants with lower levels of cognitive reappraisal (adaptive emotion regulation strategy) displayed higher orthorexia nervosa tendencies in our sample. Our finding shows that in the absence of adaptive emotion regulation strategies, individuals experience amplified psychopathological symptoms (e.g., orthorexia nervosa) in response to negative metacognitive beliefs about the uncontrollability and danger of worrying thoughts. These negative beliefs have been identified as crucial factors in eating disorders [26, 28, 81]. Actually, research has suggested that persistent negative thoughts related to food and/or body image may become central to negative self-evaluations over time [28, 79]. Consequently, individuals with eating disorders may engage in complex metacognitive processes making them regard their negative beliefs as uncontrollable and harmful [28]; which may, in turn, prompt them to indulge in compensatory and restrictive eating behaviors as a way of circumventing negative outcomes such as weight gain (or an unhealthy body in the case of orthorexia nervosa). In contrast, among people manifesting heightened levels of cognitive self-consciousness, those having low levels of expressive suppression (maladaptive emotion regulation strategy) in our sample exhibited less symptoms of orthorexia nervosa. Our finding implies that in the absence of maladaptive emotion regulation strategies, the inclination to consistently direct one’s attention towards their own cognitive processes results in no or less negative and obsessive thinking patterns related to food and eating behaviors.

Our analysis also indicated that higher physical activity was associated with more orthorexia nervosa tendencies, in line with previous literature [90]. According to some concepts, individuals exhibiting disordered eating behaviors may engage in excessive exercise as a means of avoiding or coping with their emotions [91]. Finally, our study showed that lower socio-economic status and higher educational level (university level compared to secondary level) were related to less orthorexia nervosa tendencies. Prior reports have suggested that individuals from socio-economically disadvantaged backgrounds, particularly in the context of the current economic crisis in Lebanon, may prioritize meeting basic survival needs and obtaining adequate nutrition over concerns related to their weight or the quality and healthfulness of their diets [36]. In addition, despite some evidence suggesting that people with higher levels of education may be more susceptible to exhibit orthorexia nervosa tendencies, owing primarily to greater awareness and concern about adequate nutrition and health [92], our study found an opposite relationship. Consistent with our finding, some researchers have however revealed that individuals with lower levels of education may be at greater risk for developing orthorexia nervosa [11], potentially due to limited access to reliable and accurate information about nutrition and health. Overall, further research is still necessary to gain a better understanding of the association between educational level and orthorexia nervosa, as this relationship may be influenced by a variety of factors including the type of education and university major (e.g., nutrition students may be more susceptible to orthorexia nervosa compared to other students) [93].

### Clinical implications

Dysfunctional metacognitive processes in individuals with orthorexia nervosa have yet to be explored in the literature. Through shedding light on the relationships among dysfunctional metacognitive processes, emotion regulation strategies, and orthorexia nervosa, our study has filled a significant gap in orthorexia nervosa research; which could help to better comprehend, identify, and manage this pathology. Our findings specifically endorse that comparably to individuals with other eating and psychopathological disorders, people with orthorexia nervosa may benefit from effective treatment strategies to manage dysfunctional thought patterns and negative emotions; such as metacognitive therapy, cognitive behavioral therapy, mindfulness-based interventions, or acceptance and commitment therapy; in order to enhance their awareness of their cognitive and affective states, and to foster the development of more adaptive coping strategies in response to them [94–98]. Building upon these perspectives, our study provides the groundwork for future work aiming at advancing research into the psychopathology, risk factors, and evidence-based therapeutic approaches for orthorexia nervosa.

### Limitations

The results of our study should be interpreted in light of certain limitations, such as the cross-sectional design that impedes the establishment of causal relationships between the variables. Future cohort studies are warranted to reproduce our findings through temporal frameworks. In addition, all data were collected via the snowball sampling technique, which limits the generalizability of our findings to the whole Lebanese population. Indeed, due to potential difficulties some individuals may face in accessing internet services, addressing this issue in future research could improve the study findings’ overall representativeness. A residual confounding bias is also possible; additional variables could be considered as potential mediators and moderators of the relationships between dysfunctional metacognition and orthorexia nervosa in future research. In addition, evaluations of general health, mental health, and/or eating disorders must be considered in future research, in order to control for these comorbidities and test whether participants with a history of eating disorders, for instance, would be more inclined to participate in the survey, potentially impacting the research findings.

## Conclusion

Our study provides the first empirical evidence for the existence of significant associations between dysfunctional metacognitive processes and orthorexia nervosa. It also highlights that these pathways are considerably modulated by individuals’ ability to regulate their emotions adaptively versus maladaptively. These results further support that orthorexia nervosa shares features with other eating disorders and can be treated as a particular form of feeding and eating disorders. Our findings thus suggest that therapies aimed at improving thought monitoring and emotional regulation may be beneficial for individuals with symptoms of orthorexia nervosa.

## Data Availability

All data generated or analyzed during this study are not publicly available, but are available upon a reasonable request from the corresponding author (SH).
